# The Impact of Diabetes Mellitus and Metformin Use on Outcomes After Endovascular Aneurysm Repair

**DOI:** 10.3390/jcm14010295

**Published:** 2025-01-06

**Authors:** Tara A. R. van Merrienboer, Veerle Warlich, Suzanne Holewijn, Wouter Driessen, Kak K. Yeung, Michel M. P. J. Reijnen

**Affiliations:** 1 Department of Surgery, Amsterdam UMC Location University of Amsterdam, Meibergdreef 9, 1105 AZ Amsterdam, The Netherlands; k.yeung@amsterdamumc.nl; 2 Department of Surgery, Amsterdam UMC Location Vrije Universiteit Amsterdam, De Boelelaan 1117, 1081 HV Amsterdam, The Netherlands; 3 Amsterdam Cardiovascular Sciences, Atherosclerosis & Ischemic Syndromes, 1105 AZ Amsterdam, The Netherlands; 4 Department of Surgery, Rijnstate, Wagnerlaan 55, 6815 AD Arnhem, The Netherlands; veerle.warlich@gmail.com (V.W.); shole-wijn@rijnstate.nl (S.H.); wodriessen@rijnstate.nl (W.D.); mmpj.reijnen@gmail.com (M.M.P.J.R.); 5 Multi-Modality Medical Imaging Group, TechMed Center, University of Twente, Hallenweg 5, 7522 NH Enschede, The Netherlands

**Keywords:** aortic aneurysm, abdominal, metformin, diabetes mellitus, endovascular aneurysm repair

## Abstract

**Objective**: To study the influence of diabetes mellitus (DM) and metformin treatment on aneurysm sac remodeling after endovascular aneurysm repair (EVAR). **Methods**: A retrospective single-center cohort analysis was conducted on consecutive patients who underwent elective EVAR for an infrarenal abdominal aortic aneurysm (AAA) between January 2011 and December 2021. Differences between study groups were analyzed and Kaplan–Meier analysis were employed to describe overall and reintervention-free survival. Cox regression analysis was performed to identify predictors of sac shrinkage. **Results**: A total of 529 patients were included: 74 (14.0%) had DM and metformin treatment, 26 (4.9%) had DM without metformin treatment, and 429 (81.1%) did not have DM. At one-year follow-up, diabetic patients showed significantly less sac shrinkage compared to non-diabetic patients (40.0% vs. 52.0%; *p* = 0.038), with a trend toward more stable sac behavior in diabetic patients (52% vs. 42%; *p* = 0.055). At last follow-up, sac shrinkage was significantly less in diabetic patients on metformin treatment compared to non-diabetics (48.6% vs. 59.9%; *p* = 0.047). No differences in sac shrinkage were observed between diabetics with and without metformin treatment. The presence of endoleak was significantly higher in groups showing stable sac behavior and growth. Through nine-year follow-up, overall survival was significantly less in diabetic patients compared to non-diabetic ones (23.5% vs. 37.5%; *p* < 0.001). **Conclusions**: This study showed a negative impact of diabetes mellitus and metformin treatment on sac shrinkage following EVAR. The presence of any type of endoleak was associated with reduced sac shrinkage at both time points. Overall survival was significantly lower in diabetic patients compared to non-diabetic patients.

## 1. Introduction

For patients with an abdominal aortic aneurysm (AAA), who have suitable anatomy, endovascular aneurysm repair (EVAR) is considered as the preferred approach over open repair. The shrinkage of the aneurysm sac is often used as an indicator of successful technical outcomes [1], and it is associated with fewer complications for up to five years after EVAR [2]. The factors determining whether an aneurysm will shrink or remain stable after EVAR are still mainly unknown. Several risk factors have been identified that are associated with decreased sac shrinkage, including renal impairment, type I and type II endoleaks (EL), preoperative neck thrombus, and aortic calcification [1,3].

Diabetes mellitus (DM) is a known risk factor for cardiovascular disease. However, recent studies have shown that patients with type 2 diabetes have a lower risk of the development, growth, and rupture of an abdominal aortic aneurysm (AAA) [4,5]. The mechanism of how DM may influence the development and risks of AAA remains unclear. However, recent studies have described that DM patients have a thickened wall and decelerated matrix loss, potentially due to advanced glycation end product (AGE) accumulation and lower concentrations of matrix metalloproteases (MMP) [5,6], which may contribute to the decreased risk of AAA and aneurysm growth.

Metformin is the first-line treatment for type 2 diabetes. Observational cohort studies have described smaller AAA growth rates in type 2 diabetes patients treated with metformin than those treated with alternative antidiabetic therapies, even after adjusting for other risk factors [7,8]. A recent systematic review and meta-analyses showed an estimated mean growth difference of AAA diameter between metformin users and non-users of 0.73 mm/year [9]. In animal studies, metformin attenuated AAA formation and progression [10,11]. In our recent study, we found that metformin increases contraction and metabolic activity while reducing proliferation capacity, migration, and inflammation in vitro in aortic smooth muscle cells (SMCs) derived from AAA patients [12]. The potential benefits of metformin on AAA growth are under investigation in randomized controlled trials (RCTs) [13,14,15,16].

In this study, we investigated the impact of DM, with and without metformin treatment, on aneurysm sac remodeling following elective EVAR, independent of the type of endograft used. Based on the findings of the prior studies mentioned above, we hypothesize that DM and metformin use promote stable sac behavior and inhibit sac growth after EVAR. Whether or not these physiological mechanisms also have an effect on sac shrinkage remains to be determined in this study.

## 2. Methods

### 2.1. Study Design

This observational retrospective cohort study included consecutive patients who underwent elective EVAR for an infrarenal AAA between January 2011 and December 2021 in a single tertiary referral hospital. Preoperative, procedural, and follow-up data (six to eight weeks, one-year post-EVAR, and yearly after that) are prospectively derived from the electronic health records continuously for all our AAA patients and entered coded into Research Manager (Research Manager, Deventer, The Netherlands). Preoperative data included a medical baseline assessment by the anesthesiologist, which was used to evaluate risk factors. At this timepoint, diabetes status and metformin use were specifically assessed. A waiver from the medical ethics committee (2023-16141) and approval from the local board of directors (2023-2183) were obtained. The institute’s opt-out registry was consulted to determine whether or not patients objected to participating in scientific research.

Treatment procedures were performed according to the device’s instructions for use and the local protocols. All patients were discussed in multidisciplinary meetings, and device selection was based on anatomy and the surgeon’s preference. Post-operative medical treatment consisted of statins and single anti-platelet therapy, unless oral anticoagulation was used for other indications. After discharge from the hospital, a computed tomography angiography (CTA) or duplex ultrasound scanning (DUS) was done after six to eight weeks. This was followed by imaging, either DUS or CTA, at one year and annually afterwards. Diameters were measured at the available imaging modality. When both DUS and CTA were available, CTA data were used.

### 2.2. Definitions

Primary technical success was defined on an intention-to-treat basis and required a successful introduction and deployment of the device without surgical conversion, mortality, type I or III endoleaks, or graft limb obstruction. Based on the above criteria, if unplanned endovascular or surgical procedures were required to achieve success, assisted technical success was designated as “yes” if successful and “no” if unsuccessful.

### 2.3. Outcomes

The primary objective was to determine the impact of DM and metformin treatment on aneurysm sac remodeling after elective EVAR. Sac remodeling was defined as sac shrinkage (decrease of >5 mm from baseline), stable sac behavior (change of less than or equal to 5 mm from baseline), and sac growth (an increase of >5 mm from baseline). Secondary endpoints included the presence of EL, including the type of endoleak, reintervention-free survival, overall survival, and freedom from aneurysm-related mortality, all defined according to EVAR reporting standards [17,18,19].

### 2.4. Statistical Analysis

The imputation of missing values was not applied, and percentages were calculated based on the entire study groups. Normality was tested using Shapiro–Wilk tests for all study groups. Continuous variables are presented as the mean and standard deviation (SD) when the data are normally distributed, or as the median and interquartile range (IQR) in the case of skewed data. Discrete variables are presented as absolute values followed by percentages. Differences between groups were tested using independent samples t-tests, Chi-square tests, or independent samples Mann–Whitney U tests. Bar charts were used to show the proportional distribution of sac remodeling outcomes at one year and the last follow-up. Kaplan–Meier curves were used to describe overall and reintervention-free survival. Log-rank tests were used to estimate differences between the groups. Cox regression analysis was performed to identify potential predictors of sac shrinkage. Statistical analysis was performed using IBM SPSS Statistics (SPSS version 29.0 for Windows, IBM Corporation, Armonk, NY, USA). A two-sided *p*-value < 0.050 was considered significant.

## 3. Results

**Figure 1:**
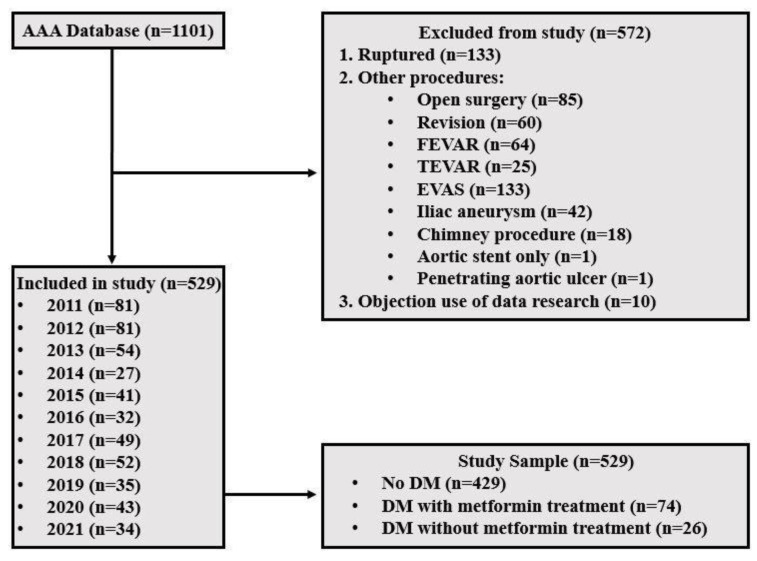
Inclusion flow chart. FEVAR—fenestrated endovascular aneurysm repair. TEVAR—thoracic endovascular aneurysm repair; EVAS—endovascular aneurysm sealing, No DM—patients without diabetes mellitus, DM—diabetes mellitus.

The database contained 1101 patients. Patients who underwent an elective repair of an infrarenal aneurysm of the abdominal aorta were included in the current study. After the exclusion process, 529 patients remained eligible for enrollment in the study, of whom 74 (14.0%) were diabetic patients with metformin therapy (DM + MF), 26 (4.9%) diabetic patients without metformin therapy (DM-MF), and 429 (81.1%) non-diabetic patients (No DM) (Figure 1). The median follow-up time until the last available follow-up was 3.8 years (IQR 1.6; 6.6 years) without significant differences between groups.

### 3.1. Baseline Patient and AAA Characteristics

Compared to the non-diabetic patients, the overall group of diabetic patients had a higher body mass index (BMI) (*p* < 0.001), higher glucose levels (*p* < 0.001), a higher ASA classification (*p* < 0.001), a higher prevalence of hypertension (HT) (*p* = 0.001) and hyperlipidemia (*p* = 0.033), and more frequently a history of cardiac disease (*p* = 0.014) (Table 1). In addition, diabetic patients without metformin treatment more often had a renal history (*p* = 0.037) compared to non-diabetic patients. Of the diabetic patients prescribed metformin, 27.0% exclusively used metformin, 66.2% used additional oral diabetes medication, and 37.8% relied on insulin next to their oral diabetes therapy. Out of the diabetic patient group without metformin prescription, 38.5% used oral DM medication and 38.5% of patients used insulin next to oral DM medication.

There were no significant differences between non-diabetic and diabetic AAA patients in the pre-operative maximum aneurysm diameter, type of aneurysm, and device used, as shown in Table 1. The infrarenal aortic neck diameter was larger, and the diameter of the right common iliac artery was smaller in the diabetic patient group without metformin compared to the non-diabetic group (26.0 mm, IQR 24.0–28.0 mm vs. 23.5 mm, IQR 21.0–25.0 mm; *p* = 0.004, and 14.0 mm, IQR 11.0–18.0 mm vs. 16.0 mm, IQR 13.0–20.0 mm; *p* = 0.050).

### 3.2. Procedure, Hospitalization, and 30-Day Complications

Primary technical success was achieved in 86.4% of cases and assisted technical success in 98.3% without differences between groups. The diabetic group overall had a longer hospital stay (3.0 days, IQR 2.0–5.5 days vs. 3.0 days, IQR 2.0–4.0 days) (*p* = 0.017), a longer procedural time (100.5 min, IQR 75.5–120.0 min vs. 88.0 min, IQR 71.5–112.0 min) (*p* = 0.039), and more complications during the hospitalization (38.0% vs. 23.1%) (*p* = 0.002) compared to the non-diabetic group. The diabetic patients without metformin had less blood loss (0.0 mL, IQR 0.0–100.0 mL vs. 100.0 mL, IQR 0.0–300.0) (*p* = 0.020) compared to the non-diabetic patients (Table 2).

### 3.3. Sac Remodeling

**Figure 2:**
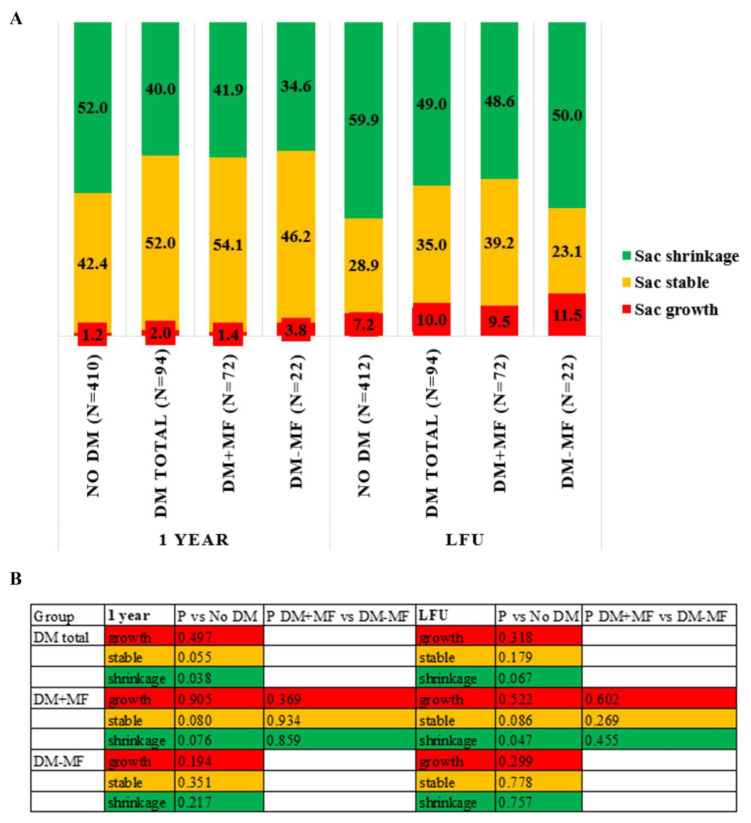
Sac remodeling at one-year and the last follow-up. (**A**) Percentage of aneurysm sac shrinkage, stability, and growth across multiple study groups at one-year follow-up and at the last follow-up. The total percentage is not equal to 100% due to missing data. (**B**) *p*-values from Pearson Chi-square tests comparing aneurysm sac outcomes (shrinkage, stability, and growth) between diabetic and non-diabetic AAA patients, as well as between diabetic patients with and without metformin treatment. No DM—patients without diabetes mellitus, DM total—patients with diabetes mellitus, DM + MF—patients with diabetes mellitus on metformin treatment, DM-MF—patients with diabetes mellitus without metformin treatment, LFU—last follow-up, P—*p*-value.

Overall, sac shrinkage was observed in 49.7% of patients at one-year follow-up and in 57.8% at the last follow-up. At one-year follow-up, 40.0% of the diabetic patients had sac shrinkage compared to 52.0% in the non-diabetic patients (*p* = 0.038). Additionally, there was a trend toward more stable sac behavior in diabetic patients compared to non-diabetic patients (52% vs. 42%; *p* = 0.055). There were no differences in shrinkage percentages between the diabetic patients with and without metformin treatment (Figure 2). At the time of the last available follow-up, the percentage of sac shrinkage was 49.0% in diabetic patients compared to 59.9% in non-diabetic patients (*p* = 0.067). Sac shrinkage was significantly less in diabetic patients using metformin compared to the non-diabetic group (48.6% vs. 59.9%; *p* = 0.047). No significant differences in sac remodeling were observed between diabetic patients with metformin as monotherapy and those not using metformin at one-year follow-up and at the last available follow-up. 

The Cox regression analysis on sac shrinkage revealed that a higher ASA group, advanced age, and a larger baseline AAA maximum diameter are associated with an increased likelihood of sac shrinkage. Conversely, the presence of hyperlipidemia, reported endoleak during follow-up, and reintervention are associated with a decreased likelihood of sac shrinkage. However, the magnitude was low (Supplementary Table S1). The type of endoprosthesis did not influence sac remodeling. None of the other aneurysm characteristics were significantly associated with sac shrinkage (Supplementary Table S2).

### 3.4. Secondary Endpoints

**Figure 3:**
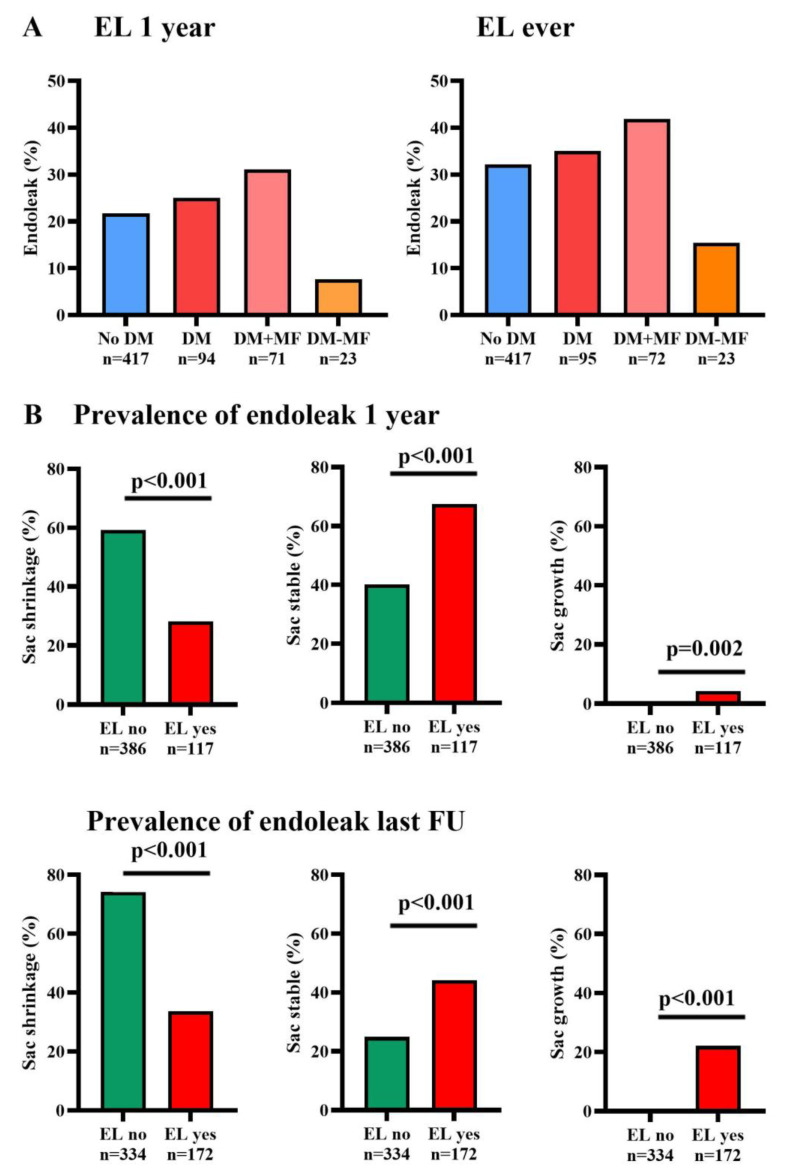
Endoleak prevalence at one-year and the last follow-up. (**A**) Prevalence of endoleak status across study groups at one-year and last follow-up, with endoleak presence shown as a percentage. (**B**) Prevalence of sac remodeling by endoleak status at one-year and last follow-up. Sac remodeling outcomes are shown as percentages within each endoleak group; *p*-values indicate the statistical significance of differences in sac remodeling outcomes between patients with and without endoleak, with comparisons based on Pearson Chi-square test. No DM—patients without diabetes mellitus, DM total—patients with diabetes mellitus, DM + MF—patients with diabetes mellitus on metformin treatment, DM-MF—patients with diabetes mellitus without metformin treatment, LFU—last follow-up, EL = endoleak, P—*p*-value.

The incidence of the first detected endoleak of any type during follow-up is depicted in Figure 3A at one year and the last available follow-up. Overall, at one-year follow-up, 22.3% and at the last follow-up, 32.7% of patients had an endoleak of any type. There were no significant differences between groups, and in all groups the incidence increased over time, as depicted in Figure 3A.

Figure 3B shows the prevalence of sac remodeling outcomes according to endoleak status at one year and last follow-up. At both time points, the presence of an endoleak was significantly higher than its absence in the stable and growing sac behavior groups (*p* < 0.002). In contrast, the prevalence of an endoleak was significantly lower in the shrinking aneurysm group compared to the absence of an endoleak (*p* < 0.001 for both time points). The first detected endoleak types per subgroup are available in Supplementary Table S3.

We restricted our comparison of reintervention-free survival to the five-year follow-up, as the number of patients in the diabetic group was fewer than ten beyond that point. At the five-year follow-up, reintervention-free survival was 75.7% (95% CI: 5.4–6.5) for diabetic patients and 81.1% (95% CI: 8.5–9.4) for non-diabetic patients (*p* = 0.355; see Supplementary Figure S1). No significant differences were observed between the subgroups. A total of 100 patients (18.9%) needed a reintervention during follow-up, of which 38 (7.2%) were within the first year of follow-up, without significant differences between subgroups. A total of 75 patients required a single reintervention. Additionally, some patients needed multiple reinterventions: 13 patients underwent two reinterventions, eight required three, three needed four, and one patient underwent five reinterventions. The median time to first reintervention was 25 months (IQR 2.5–58.7 months). The median time until the first reintervention was 5.3 months (IQR 1.5–50.9 months) for diabetic patients compared to 26.5 months (IQR 3.0–61.8 months) for non-diabetics (*p* = 0.295).

**Figure 4:**
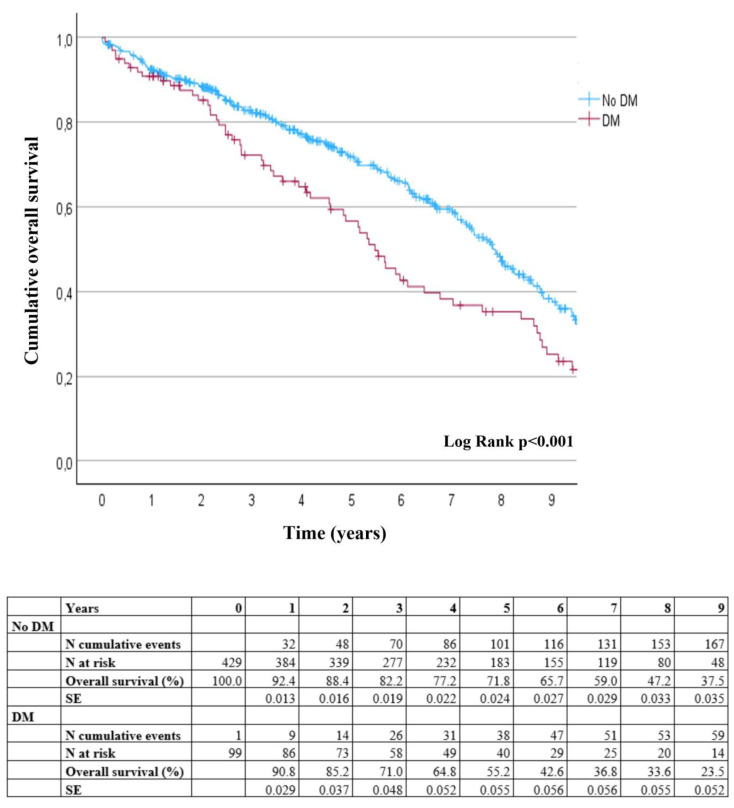
Kaplan–Meier overall survival curves. Overall survival during nine years of follow-up after endovascular aneurysm repair comparing non-diabetic patients with diabetic patients. No DM—patients without diabetes mellitus, DM total—patients with diabetes mellitus, SE—standard error, *p* denotes the overall *p*-value of patients with diabetes with and without metformin treatment compared to patients without diabetes.

The overall survival at one-year follow-up was 90.8% (95% CI: 29.1–32.9) for diabetic patients versus 92.4% (95% CI: 36.0–37.9) for non-diabetic patients (*p* = 0.574). We limited the Kaplan–Meier analysis until nine years of follow-up because the number of patients in the diabetic group decreased beyond ten at that point. Through nine-year follow-up, overall survival was 23.5% (95% CI: 4.7–6.2) for diabetic patients, compared to 37.5% for non-diabetic patients (95% CI: 7.3–8.4) (*p* < 0.001) (Figure 4). At the median follow-up of 3.8 years, 46.7% (*n* = 247) of the patients had passed away. Aneurysm-related deaths occurred in five patients: four patients without diabetes and one patient with diabetes. Among the non-diabetic patients, aneurysm-related deaths occurred on the day of the procedure (one patient), at four days post-procedure (two patients), and at five days post-procedure (one patient). The diabetic patient experienced an aneurysm-related death 50 days after the procedure.

## 4. Discussion

This observational retrospective cohort study revealed less sac shrinkage at one-year follow-up after elective EVAR in diabetic patients than in non-diabetic patients, although there was a trend toward more stable sac behavior in diabetic patients. At the last follow-up available, the difference in sac shrinkage was still 10.9% in favor of the non-diabetic group, but this difference did not reach statistical significance. However, at the last follow-up, diabetic patients using metformin showed a significantly lower percentage of sac shrinkage compared to the non-diabetic group. Moreover, the presence of endoleaks increased over time, correlating with diminished sac shrinkage at both time points. Overall survival was significantly reduced among diabetic patients, but there were no significant differences in reintervention-free survival and freedom from aneurysm-related mortality across the groups.

A negative relationship between type 2 diabetes and AAA prevalence, growth, and rupture has been previously described [4,5]. However, studies investigating the influence of DM on postoperative outcomes after EVAR have shown diverse results.

Focusing on early outcomes after EVAR, we observed that DM was associated with prolonged hospitalization, extended operative durations, and an increased incidence of complications during hospitalization. Inadequate controlled blood glucose is known to contribute to worse surgical outcomes, including a higher incidence of infections, delayed wound healing, increased mortality, and longer hospital stays [20]. These findings underscore the critical role of effective glycemic management during the perioperative phase of EVAR.

A nationwide Swedish observational cohort study, comparing 748 patients with DM and 2630 without, using propensity score-adjusted analysis, found no difference in overall or cardiovascular mortality. Yet, there was a reduced need for reintervention and higher rates of acute myocardial infarction in the DM group during the four-year follow-up. However, this study did not consider the sac remodeling (including growth, stable behavior, and shrinkage) and amount and type of endoleaks [21]. In another study, DM patients had decreased rates of sac growth after EVAR during four years of follow-up, and they found a trend towards a lower need for reintervention. In the same study, they found no differences in mortality rate or endoleak occurrence between diabetic and non-diabetic patients [22]. A recent study evaluated the sac remodeling during five years of follow-up after an EVAR in diabetic and non-diabetic patients treated with a Gore excluder endograft. This study found no difference in sac regression rates across the groups, only a higher five-year mortality rate in diabetic patients, even adjusted for other comorbidities [23]. Moreover, a meta-analysis aimed at identifying and evaluating factors influencing sac shrinkage after EVAR found that DM was associated with a trend toward reduced sac shrinkage [1].

The studies mentioned above exclusively examined the effects of DM and did not consider the influence of metformin usage. Our study found less sac shrinkage at the last follow-up, with a median of 3.8 years, in diabetic patients using metformin compared to non-diabetic patients. However, the number of patients in the diabetic group not using metformin is relatively low. In contrast, a comparable single-center study reported that metformin use does not seem to influence long-term post-EVAR AAA sac remodeling [24]. Due to the diverse results of the effect of diabetes and metformin use on post-EVAR outcomes, including sac remodeling and overall survival, more studies are needed.

Based on the studies above, it appears that patients with diabetes may experience reduced sac growth over time; however, there is also no evidence of a beneficial effect of DM on sac shrinkage following EVAR. Multiple changes observed in the aortic wall of diabetic patients may partially explain this phenomenon. Patients with AAA have a reduction in the aortic wall matrix, whereas individuals with DM often have an increase in the vascular matrix [25]. Furthermore, collagen synthesis is increased in DM, which increases aortic wall thickness and decreases levels of matrix metalloproteinases (MMPs), which degrade the extracellular matrix (ECM). In addition, hyperglycemia and hyperinsulinemia both occur in DM, causing an increase in advanced glycation end products (AGEs). AGEs bind the collagen and elastin of the aortic wall and promote vascular SMC proliferation, leading to increased resistance and strength of the aortic wall [26]. Subsequently, this leads to a decreased risk of AAA. However, the stiffness of the aortic wall can also be responsible for the fact that we see less sac shrinkage and a trend towards more stable sac behavior in diabetic patients compared to non-diabetic patients post-EVAR.

The prevalence of hyperlipidemia was higher in diabetic patients compared to non-diabetic patients, and in the Cox regression analysis hyperlipidemia was negatively associated with sac shrinkage, although the magnitude was low. Other baseline differences in comorbidities, such as hypertension, body mass index, cardiac history, and glucose levels before the operation, were not significant predictors of sac shrinkage in our analysis of the overall cohort. Furthermore, consistent with our findings, a large meta-analysis of patients undergoing endovascular aneurysm repair also found no association between comorbidities such as hypertension, obesity, and coronary artery disease with postoperative sac shrinkage [1]. Notably, this meta-analysis showed that hypercholesterolemia had a positive impact on sac shrinkage, and there was also a trend toward an association between sac shrinkage and statin therapy. Therefore, the higher incidence of these comorbidities in the diabetes mellitus group may not fully explain the reduced sac shrinkage observed.

Metformin is suggested to affect AAA progression [9], and multiple RCTs have been initiated to investigate this effect in patients with a small aneurysms. In animal studies, the use of metformin was linked to a decrease in AAA formation and progression, alongside the preservation of medial elastin and aortic SMCs, a reduction in the accumulation of immune cells, and decreased levels of neovascularization [10,11]. It could be that these effects of metformin are beneficial against aneurysm growth, but these effects are not necessarily advantageous for aortic shrinkage. At one-year and at last follow-up, the sac growth was less, and the percentage of stable sac behavior was higher in the diabetic group using metformin than in the diabetic group without metformin treatment. However, these differences were not statistically significant. Furthermore, in a recent study, diabetic patients were more likely to have stable sac behavior, whereas non-diabetics were more likely to have sac regression at one-year follow-up after EVAR [27]. In addition, they found that diabetic patients treated with non-insulin anti-diabetic medications had a lower risk for rupture after EVAR.

In our study, the presence of endoleaks, regardless of type, increased over time and was correlated with decreased sac shrinkage at both one year and the last follow-up. Additionally, in the Cox regression analysis, the presence of endoleaks was significantly associated with reduced sac shrinkage. This is in line with the results of a previous meta-analysis in which the presence of an endoleak was correlated with less sac shrinkage post-EVAR [1]. These findings underscore the importance of vigilant monitoring and management strategies for endoleaks during follow-up.

We observed a significant reduction in overall survival over a follow-up period of nine years among diabetic patients, regardless of metformin use, compared to non-diabetic patients. This observation is consistent with a prior study that matched diabetic patients with non-diabetic controls of similar age and comorbidities [23], a recent large observational study with an eight-year follow-up [27], and a meta-analysis that included 12 cohort studies with a total of 20,210 patients who underwent AAA repair [28]. However, these results conflicted with findings from other studies in which diabetes had no impact on overall mortality [21,22]; these studies had shorter follow-up periods, and in the first few years after EVAR in our study we also observed no difference in mortality. Given that there was no difference in reintervention-free survival or freedom from aneurysm-related mortality between diabetic and non-diabetic patients, this highlights the importance of focusing on effective glycemic management and controlling other comorbidities, such as optimizing cardiovascular risk management in diabetic patients, to improve overall survival.

### Study Limitations

This study has several methodological limitations that need to be acknowledged. First, the diameters were measured using the available imaging modalities, either CTA or DUS. DUS measurements cannot be directly compared to those from CT. However, the difference in the measurement of the mean diameter of the aorta in aneurysmal patients is less than 5 mm [29]. As the latest guideline recommends, the initial scan 30 days post-op was a CTA, and patients who have undergone EVAR are recommended for long-term imaging follow-up [30]. At our institution, DUS was the preferred imaging modality for long-term follow-up. Consequently, the same modality was used for most scans from one year post-op until the last follow-up.

Additionally, this retrospective study is based on a manually entered database, which introduces the possibility of human error and missing data. The duration from the onset and diagnosis of diabetes and the initiation of metformin therapy, prior to inclusion, was unknown. These factors may have influenced the outcomes and would be interesting to explore in future research.

Another limitation lies in the significant differences in sample sizes between subgroups, particularly in the diabetic group that did not receive metformin. The smaller number of patients at risk during longer follow-up periods impacted the normality and variance assumptions. While non-parametric tests addressed this issue, the statistical power was nonetheless reduced. Furthermore, the large variance in follow-up periods complicated the time analysis up to the last follow-up; however, the follow-up periods did not differ between the study groups. Moreover, only a small percentage of diabetic patients in the study used metformin as a monotherapy. No significant differences in sac remodeling were observed between diabetic patients with metformin as monotherapy and those not using metformin at both time points. Because of the small group using metformin as monotherapy, we chose to distinguish between diabetic patients using metformin and those not using metformin. The use of other diabetic medications, such as sulfonylurea derivatives and insulin, may have influenced the results. However, we also tested the use of sulfonylurea derivatives in a Cox regression analysis on sac shrinkage as a potential predictor, but this was not significant. On the other hand, previous observational studies have only shown a significant effect of metformin on AAA growth [7,8]. We acknowledge that it would be better to report HbA1c levels rather than glucose levels to reflect long-term glucose control. Nevertheless, HbA1c data were only available for half of the patients. In addition, multiple significantly different predictors at baseline impacted sac remodeling and overall survival. Lastly, the fact that the study spanned a period of ten years could also have influenced surgical outcomes, as increased experience over time may have affected the success rate of the procedures.

## 5. Conclusions

This retrospective study showed a negative impact of diabetes mellitus and metformin use on sac shrinkage following EVAR. Diabetic patients showed significantly less sac shrinkage than non-diabetic patients at one-year follow-up. However, at this time point there was a trend towards more stable sac behavior in the diabetic patients. At the last follow-up, only those using metformin had lower sac shrinkage percentages compared to the non-diabetic group. Additionally, the presence of an endoleak increased over time in both non-diabetic and diabetic AAA patients, correlating with diminished sac shrinkage at both time points. These findings emphasize the importance of careful monitoring and management strategies for endoleaks during follow-up. Overall survival was significantly reduced among diabetic patients compared to non-diabetic patients. Furthermore, the groups had no significant differences in reintervention-free survival and freedom from aneurysm-related mortality. Further prospective and multicenter studies are needed to clarify the effects of diabetes and metformin use on post-EVAR outcomes, due to the varied results in the literature on this topic.

## Figures and Tables

**Table 1:** Baseline characteristics per subgroup.

	No DM (*n* = 429)	DM Total (*n* = 100)	*p*	DM + MF (*n* = 74)	*p*	DM-MF (*n* = 26)	*p*
Age (years)	72.8 ± 8.1	73.0 ± 0.82	0.852	72.2 ± 7.7	0.551	75.2 ± 9.3	0.164
Male sex	361 (84.1)	89 (89.0)	0.220	65 (87.8)	0.416	24 (92.3)	0.263
BMI (kg/m^2^)	25.8 (23.8; 28.4)	27.81 (24.6; 31.0)	<0.001	27.8 (24.8; 31.0)	<0.001	27.6 ± 4.5	0.058
SBP (mmHg)	143.0 (127; 156.5)	143.06 (127.5; 160.0)	0.596	143.0 (125.0; 160.0)	0.761	143.0 (130.0; 156.0)	0.557
DBP (mmHg)	81.0 (74; 89)	81.06 (73.0; 87.0)	0.348	81.0 (74.0; 87.0)	0.410	82.0 (70.0; 87.0)	0.599
Heart Rate (bpm)	72.5 (64; 84.5)	74 (66.0; 85.0)	0.188	73.0 (66.0; 85.0)	0.471	78.0 (70.0; 85.0)	0.134
ASA classification ≤ 2	195 (46.9)	26 (26.0)	<0.001	22 (29.7)	0.025	4 (15.4)	0.002
ASA classification ≥ 3	221 (53.1)	73 (73.0)		51 (68.9)		22 (84.6)	
Hypertension	293 (69.3)	86 (86)	<0.001	62 (83.8)	0.011	24 (92.3)	0.012
Hyperlipidemia	295 (76.8)	80 (87.0)	0.033	59 (79.7)	0.011	21 (80.8)	0.105
Cardiac history	176 (46.0)	56 (60.2)	0.014	43 (58.1)	0.017	13 (50.0)	0.324
Pulmonary history	79 (21.1)	24 (26.4)	0.279	20 (27.0)	0.115	4 (15.4)	0.602
Renal history	110 (26.3)	34 (35.4)	0.073	23 (31.1)	0.321	11 (42.3)	0.037
Tobacco use ^a^	142 (35.0)	36 (37.1)	0.692	27 (36.5)	0.680	9 (34.6)	0.917
DM type 2, controlled by diet or oral agents	-	77 (77.0)		60 (81.1)		17 (65.4)	
DM type 2, insulin-controlled ^b^	-	21 (21.0)		14 (18.9)		7 (26.9)	
DM type 1	-	2 (2.0)		-		2 (7.7)	
Glucose (mmol/L) ^c^	5.7 (5.2; 6.3)	9.1 (6.5; 11.0)	<0.001	9.2 (6.5; 11.4)	<0.001	8.8 (6.7; 10.4)	<0.001
Metformin	-	74 (76.0)	<0.001	74 (100)	<0.001	-	-
Other Oral DM medication ^d^	-	60 (66.7)	<0.001	49 (66.2)	<0.001	10 (38.5)	<0.001
Insulin ^e^	-	38 (42.2)	<0.001	28 (37.8)	<0.001	10 (38.5)	<0.001
Metformin only	-	20 (22.2)	<0.001	20 (27.0)	<0.001	-	-
AAA characteristics							
Maximum aneurysm diameter (mm)	57.0 (53.0; 63.0)	56.5 (53.2; 62.7)	0.823	57.0 (54.0; 63.0)	0.794	55.0 (53.0; 59.0)	0.350
Infrarenal aortic neck diameter (mm)	23.5 (21.0; 25.0)	24.0 (22.0; 26.0)	0.320	23.0 (21.0; 25.0)	0.696	26.0 (24.0; 28.0)	0.004
Infrarenal aortic neck length (mm)	27.5 (19.0; 37.0)	29.5 (20.0; 40.0)	0.212	30.0 (21.0; 38.0)	0.232	29.0 (16.0; 50.5)	0.610
Angle between AAA and neck (degrees)	41.5 (27.0; 60.0)	35.0 (23.0; 44.5)	0.052	30.0 (23.0; 49.0)	0.080	40.0 (26.0; 40.0)	0.327
Right CIA diameter (mm)	16.0 (13.0; 20.0)	16.0 (13.0; 19.5)	0.986	17.0 (14.0; 22.5)	0.227	14.0 (11.0; 18.0)	0.050
Left CIA diameter (mm)	15.0 (13.0; 19.0)	15.0 (13.0; 18.0)	0.679	16.0 (14.0; 21.0)	0.099	13.05 (12.0; 15.0)	0.064
Type of aneurysm			0.749		0.618		0.831
Saccular	39 (9.7)	10 (10.8)		8 (10.8)		2 (7.7)	
Fusiform	365 (90.3)	83 (89.2)		61 (82.4)		22 (84.6)	
Device type			0.463		0.338		0.868
Medtronic Endurant	241 (56.3)	55 (55.0)		40 (54.1)		15 (57.7)	
Gore Excluder	134 (31.3)	32 (32.0)		26 (35.1)		6 (23.1)	
Endologix AFX	21 (4.9)	4 (4.0)		-		4 (15.4)	
Other	32 (7.5)	9 (9.0)		8 (10.8)		1 (3.8)	

No DM—patients without diabetes mellitus, DM-total—patients with diabetes mellitus, DM + MF—patients with diabetes mellitus on metformin treatment, DM-MF—patients with diabetes mellitus without metformin treatment, BMI—body mass Index; SBP—systolic blood pressure; DBP—diastolic blood pressure; ASA—American Society of Anesthesiologists; DM—diabetes mellitus; AAA—abdominal aortic aneurysm, CIA—common iliac artery, *p*—*p*-value compared to no diabetes. Values are presented as mean ± SD, frequency (percent), or median (Q1; Q3). ^a^ Current tobacco use includes abstinence for less than 1 year. ^b^ Diabetes Type 2 patients who use only insulin to control the glucose levels. ^c^ Glucose or glucose sober is measured at baseline. ^d^ Includes sulfonylurea derivatives: Gliclazide, Glimepiride, and Tolbutamide. ^e^ Includes following insulin types: glargine, isophane, detemir, aspart/protamine/novorapid, glulisine, and lispro.

**Table 2:** Hospitalization and 30-day complication details by subgroup.

	No DM (*n* = 429)	DM Total (*n* = 100)	*p*	DM + MF (*n* = 74)	*p*	DM-MF (*n* = 26)	*p*
Time hospitalization (days)	3.0 (2.0; 4.0)	3.0 (2.0; 5.5)	0.017	4.0 (2.0; 6.0)	0.016	3.0 (2.0; 5.0)	0.425
Blood loss (mL)	100.0 (0.0; 300.0)	100.0 (0.0; 250.0)	0.719	150.0 (5.0; 300.0)	0.371	0.0 (0.0; 100.0)	0.020
Total procedure time (min)	88.0 (71.5; 112.0)	100.5 (75.5; 120.0)	0.039	98.0 (76.0; 124.0)	0.039	102.5 (75.0; 114.0)	0.474
Technical success	369 (86.0)	88 (88.0)	0.602	69 (93.2)	0.087	19 (73.1)	0.071
Assisted technical success	421 (98.1)	99 (99.0)	0.547	74 (100.0)	0.236	25 (96.2)	0.481
Conversion to open repair	1 (0.2)	1 (3.0)	0.632	1 (1.4)	0.683	0 (0.0)	0.812
Complications during hospitalization	99 (23.1)	38 (38.0)	0.002	27 (36.5)	0.011	11 (42.3)	0.026
Systemic complications	31 (31.1)	8 (21.0)		4 (14.8)		4 (36.4)	
Urine tract infection	2 (2.0)	1 (2.6)		1 (3.7)		-	
Renal function deterioration	7 (7.1)	1 (2.6)		-		1 (9.1)	
Fever	20 (20.2)	5 (13.2)		3 (11.1)		2 (18.2)	
Pulmonary complications	13 (13.5)	5 (13.2)	0.499	4 (14.8)	0.250	1 (9.1)	0.643
Cardiac complications	19 (19.8)	7 (18.4)	0.505	6 (23.1)	0.872	1 (9.1)	0.302
Angina (NYHA class)	1 (1.0)	-		-		-	
Myocardial infarction	3 (3.0)	1 (2.6)		-		1 (9.1)	
Coronary artery disease	1 (1.0)	-		-		-	
Atrial fibrillation	5 (5.1)	-		-		-	
Acute heart failure	2 (2.0)	1 (2.6)		1 (3.7)		-	
Procedure related deaths	10 (2.3)	-		-		-	

No DM—patients without diabetes mellitus; DM-total—patients with diabetes mellitus; DM + MF—patients with diabetes mellitus on metformin treatment; DM-MF—patients with diabetes mellitus without metformin treatment. *p*—*p*-value compared to no diabetes. Values are presented as mean ± SD, frequency (percent), or median (Q1; Q3).

## Data Availability

The raw data supporting the conclusions of this article will be made available by the authors upon request and can only be shared anonymously. This restriction is due to hospital policies, which prohibit data sharing without clearly defined purposes. Data transfer agreements must be in place before data can be shared.

## Supplementary Materials

The following supporting information can be downloaded at: https://www.mdpi.com/article/10.3390/jcm14010295/s1. Table S1. Results of Cox regression analysis on sac shrinkage; Table S2. Results of Cox regression analysis on sac shrinkage for aneurysm characteristics only; Table S3. Initial endoleak type by subgroup; Figure S1. Kaplan–Meier reintervention-free survival curves.
