# Supplementary material for: The Impact of Diabetes Mellitus and Metformin Use on Outcomes After Endovascular Aneurysm Repair
Source: J Clin Med. 2025 Jan 6;14(1):295. doi: 10.3390/jcm14010295 (PMC11721816; doi:10.3390/jcm14010295)
Supplement: Supplementary file 1 [file jcm-14-00295-s001.zip › jcm-3343587-supplementary.pdf]

Table S1. Results of Cox Regression analysis on sac shrinkage

	B	SE	Wald	df	Sig.	Exp(B)	95.0% CI for Exp(B)	
							Lower	Upper
ASA-group (0-2)	.505	.138	13.321	1	0.000	1.658	1.26	2.17
Age (years)	.034	.009	13.857	1	0.000	1.035	1.02	1.05
Hyperlipidemia (no)	-.468	.159	8.633	1	0.003	0.626	0.46	0.86
AAA max diameter (mm)	.027	.007	16.236	1	0.000	1.027	1.01	104
Endoleak reported during follow-up (no)	-.493	.172	8.203	1	0.004	0.611	0.44	0.86
Reintervention (no)	-.944	.227	17.345	1	0.000	0.389	0.25	0.61
Cardiac history at baseline (no)	-.242	.137	3.117	1	0.078	0.785	0.60	1.03

ASA – American Society of Anesthesiologists; AAA – abdominal aortic aneurysm. Variable included in the first analysis were: age, sex, ASA-group, reintervention, prosthesis type, cardiac history, endoleak ever, renal disease, hyperlipidemia, hypertension, BMI, glucose level, use of sulfonylurea derivatives, and baseline AAA diameter.

Table S2. Results of Cox Regression analysis on sac shrinkage for aneurysm characteristics only

	B	SE	Wald	df	Sig.	Exp(B)	95.0% CI for Exp(B)	
							Lower	Upper
Type of aneurysm (fusiform)	-.805	.631	1.627	1	0.202	.447	.13	1.54
Symptomatic aneurysm (no)	-.389	.888	.192	1	0.662	.678	.12	3.86
Infrarenal aortic neck diameter (mm)	-.111	.061	3.307	1	0.069	.895	.79	1.01
Infrarenal aortic neck length (mm)	-.008	.016	.260	1	0.610	.992	.96	1.02
Angle between AAA and neck (degrees)	-.015	.192	2.381	1	0.123	.985	.97	1.00

AAA – abdominal aortic aneurysm.

Table S3. Initial endoleak type by subgroup

First diagnosed endoleak type	No DM	DM total	P	DM + MF	P	DM - MF	P
	N (%)	N (%)	0.741	N (%)	0.550	N (%)	0.452
Type 1a	14 (3.3)	3 (3.0)		2 (2.7)		1 (3.8)	
Type 1b	8 (1.9)	3 (3.0)		3 (4.1)			
Type 2 IMA	9 (2.1)	5 (5.0)		4 (5.4)		1 (3.8)	
Type 2 lumbar	26 (6.1)	2 (2.0)		2 (2.7)			
Type 2 SMA	2 (0.5)						
type 2 unknown	49 (11.4)	13 (13.0)		12 (16.2)		1 (3.8)	
Type 3	3 (0.7)	1 (1.0)					
type unknown	27 (6.3)	8 (8.0)		8 (10.8)			
Total	138 (32.2)	35 (35.0)		32 (42.1)		3 (12.5)	

No DM – patients without diabetes mellitus; DM-total – patients with diabetes mellitus; DM+MF – patients with diabetes mellitus on metformin treatment; DM-MF – patients with diabetes mellitus without metformin treatment.
P – p-value compared to no diabetes.

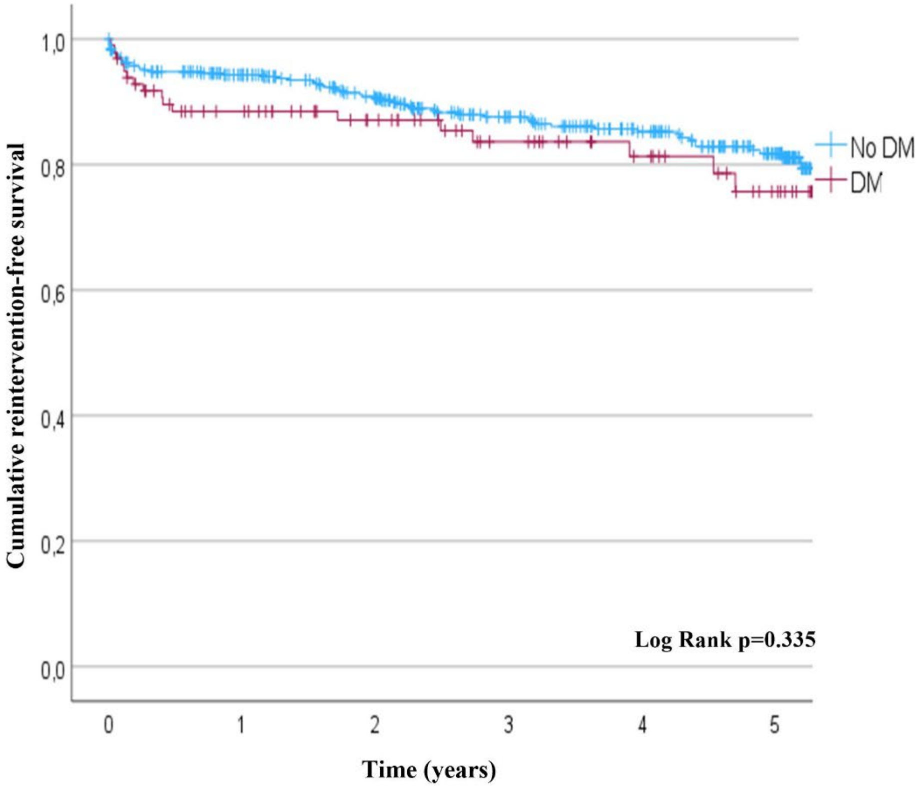

	Years	0	1	2	3	4	5
No DM							
	N cumulative events		24	37	46	52	59
	N at risk	428	357	301	238	194	134
	Overall survival (%)	100	94,3	90,5	87,6	85,2	81,8
	SE		0,011	0,015	0,017	0,019	0,023
DM							
	N cumulative events		11	12	14	15	17
	N at risk	98	74	59	43	33	21
	Overall survival (%)	100	88,5	87,1	83,6	81,3	75,7
	SE		0,033	0,035	0,041	0,046	0,058

Figure S1. Kaplan-Meier reintervention-free survival curves

Reintervention-free survival during five years of follow-up after endovascular aneurysm repair comparing non-diabetic patients and diabetic patients.

No DM – patients without diabetes mellitus, DM total – patients with diabetes mellitus, SE – standard error, P denotes the overall P-value of patients with diabetes with and without metformin treatment compared to patients without diabetes.
